# Self-assembly of multilevel branched rutile-type TiO_2_ structures via oriented lateral and twin attachment

**DOI:** 10.1038/srep24216

**Published:** 2016-04-11

**Authors:** Vanja Jordan, Uroš Javornik, Janez Plavec, Aleš Podgornik, Aleksander Rečnik

**Affiliations:** 1 Department for Nanostructured Materials, Jožef Stefan Institute & Jožef Stefan International Postgraduate School, Jamova cesta 39, SI-1000 Ljubljana, Slovenia; 2 Slovenian NMR centre, National Institute of Chemistry, Hajdrihova 19, SI-1000 Ljubljana, Slovenia; 3 Centre of Excellence for Biosensors, Instrumentation and Process Control, Tovarniška 26, SI-5270 Ajdovščina, Slovenia

## Abstract

Recent breakthrough of novel hierarchic materials, orchestrated through oriented attachment of crystal subunits, opened questions on what is the mechanism of their self-assembly. Using rutile-type TiO_2_, synthesized by hydrothermal reaction of Ti(IV)-butoxide in highly acidic aqueous medium, we uncovered the key processes controlling this nonclassical crystallization process. Formation of complex branched mesocrystals of rutile is accomplished by oriented assembly of precipitated fibers along the two low-energy planes, *i.e.* {110} and {101}, resulting in lateral attachment and twinning. Phase analysis of amorphous material enclosed in pockets between imperfectly assembled rutile fibers clearly shows harmonic ordering resembling that of the adjacent rutile structure. To our understanding this may be the first experimental evidence indicating the presence of electromagnetic force-fields that convey critical structural information through which oriented attachment of nanocrystals is made possible.

The key issue of the emerging renewable energy technologies nowadays, is fabrication of highly efficient hierarchic/branched materials with large specific surface area, increased capacity, adaptable morphology and surface properties that enable short electron and mass transport pathways, tailored for diverse applications^1^. In response to these challenges material’s scientists have begun exploring non-classical crystallization routes that involve the use of organic templates and electrostatic interactions that assist self-organization of crystalline matter into desired architectures. Bottom-up design of hierarchic 3D nanostructures requires control over the nucleation and assembly of first precipitates^2^,^3^,^4^. Understanding these mechanisms is essential to tailor chemical and physical properties of self-assembled materials. While the control over the crystallization process is needed to design crystal shape and morphology, oriented attachment provides mechanism for spontaneous self-assembly of branched and mesocrystal structures with a high degree of crystallographic registry between the constituent parts^5^. Self-assembly of nanoparticles is generally coupled with changing chemical conditions that drive surface potential to approach the isoelectric point^6^,^7^,^8^. This, in effect, causes a reduction of electrostatic barriers and promotes fast and irreversible aggregation of the primary particles^9^. With this process the total energy of the system is decreased by the surface energy reduction associated with unsatisfied surface bonds^4^,^10^, while dipole fields that exist around the primary particles are considered responsible for the unique hierarchical assembly of mesocrystals^3^,^4^,^11^,^12^. Depending on the synthesis conditions the primary particles can align to each other with such a perfection that resulting mesocrystals exhibit scattering patterns resembling that of a single crystal^4^,^5^,^13^. There is virtually no material that would be unsuitable for growing hierarchic superstructures via self-assembly, as long as the surfaces of the attaching particles are dimensionally similar^10^. Here, solution methods, *e.g.* hydrothermal crystallization procedures are becoming increasingly attractive because of their low growth temperature, low cost, and a simple scale-up^1^,^4^.

One of the most thoroughly studied materials that exhibits self-assembly and twinning, necessary for producing hierarchic fractal-like branched structures, is rutile-type TiO_2_^14^,^15^,^16^,^17^,^18^,^19^,^20^. Moreover, the rutile phase exhibits an exceptional combination of electronic and optical properties, including appropriate band-gap structure, thermodynamic stability and chemical resistance^14^,^21^,^22^,^23^,^24^,^25^,^26^,^27^, which makes it adaptable for a range of applications, such as: photocatalysis^28^,^29^,^30^,^31^, photovoltaics^32^,^33^,^34^,^35^,^36^, sensors^37^,^38^, *etc*. Branched structures of rutile are generally obtained in an acidic medium with the use of different metalorganic titanium precursors^15^,^16^,^17^,^18^,^19^,^32^,^33^,^34^,^35^,^36^. Due to increased specific surface area, branched rutile nanostructures show enhanced efficiency in photo electrochemical production of hydrogen and improved solar energy conversion efficiency when compared to simple rutile nanorods^30^,^32^,^36^.

Recently, much attention is paid to the mechanisms that cause branching of rutile, being attributed to different phenomena, such as: anatase mediated branching^15^,^17^,^19^, heteronucleation^16^,^17^, acid-assisted surface corrosion^34^,^39^, oriented attachment^10^,^40^, coalescence twinning^40^, and crystal splitting^33^,^41^,^42^. Common to these studies is that branching of rutile appears to be inversely proportional to the acidity of the medium; where in less acidic medium the hydrolysis of the titanium (IV) precursor is believed to be more intense^16^, leading to forceful nucleation and formation of rutile microspheres, whereas under more extreme acidic conditions branched structures are evolved. The branching has been described by different attachment/twinning laws, including {111}^16^, {200}^18^, {110}^18^,^29^, {101}^17^,^18^,^19^,^20^,^43^,^44^,^45^, and {301}^44^,^45^, out of which the most important element of branching is twinning. It involves simple geometric operations that bring two or more crystals of the same phase into special orientations that are not possible by random intergrowths. In rutile, twinning is a result of topotaxial reactions^46^,^47^,^48^, deformation^47^, or attachment^18^,^19^,^40^,^49^. Following only one twining law, *e.g.* {101}, branches in eight unique directions can be produced on the primary rutile crystal^43^. These can be additionally twinned into complex fractal-like structures. Hypothetic multiple twinning and formation of multilevel fractal-like branched crystal clusters is illustrated in Fig. 1. While twinning is often reported in branched rutile structures, its origin is yet to be explained.

Until the present day there is no direct evidence on the origin of self-assembly and twinning of rutile. We designed experiments that helped us to identify the key processes during the formation of branched rutile structures and estimate the role of oriented attachment in crystal growth and twinning that would provide principal protocol for tailoring desirable branched morphologies (aspect ratio, density of branching, multilevel branching, *etc*.). With this in mind we studied the initial nucleation stages of crystal assembly and showed that under acidic hydrothermal (HT) conditions rutile crystals growth via oriented attachment of precipitated rutile fibers on {110} and {101} crystal planes, forming complex branched mesocrystal structures.

## Results

Experimental work has been planned to identify the mechanisms that control growth and branching of rutile crystals. Foremost attention was dedicated to resolving the initial stages of crystallization. First, proton nuclear magnetic resonance (^1^H-NMR) analysis of the starting solution, before HT treatment was carried out to resolve which of the two processes, hydrolysis or condensation reaction^16^,^50^, is rate determining. Aggregation of precipitates nucleated after short time of HT processing was studied by transmission electron microscopy (TEM).

During HT processing the following overall reaction between Ti(IV)-butoxide (precursor) and water (reactant) takes place:


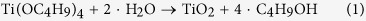


where HCl is used as a crystallization inhibitor^50^. Different HT conditions, *i.e.* precursor and reactant concentrations with acidity (OH^−^/H^+^ ratio, *see* Supporting Information; Table S1 for detailed calculations), synthesis temperature and time, were studied to understand their role in final product morphology. Depending on the choice of experimental conditions, variety of products, ranging from nanocrystal fibers and rods, twinned (branched) crystal clusters, radiating crystal clusters and microspheres composed of multiple rutile crystals radiating from the common center are synthesized (*see* Supporting Information; Figure S1).

### Hydrolysis

^1^H-NMR was used to verify whether the hydrolysis takes place before the HT synthesis, when Ti(IV)-butoxide is exposed to HCl. Characteristic chemical shifts of alkyl groups bound to titanium cation are nicely distinguishable (denoted by a, b, c and d in Fig. 2a). Sharp signal at around *δ* = 4.8 ppm corresponds to D_2_O reference. However, when we introduce 4.4 M HCl, Ti(IV)-butoxide fingerprint (Fig. 2a) is replaced by chemical shifts corresponding to free n-butanol (Fig. 2b), indicating that the hydrolysis step is completed already at room temperature (RT). This was confirmed by measuring ^1^H-NMR spectrum of n-butanol reference (Fig. 2c). Broad chemical shift at ~6.1 ppm corresponds to H_2_O in the form of OH^−^ and H_3_O^+^ groups.

### First precipitate

In the initial stages of synthesis, that are met under low reactant concentrations, high medium acidity (low OH^−^/H^+^ ratio), short processing times and low target temperature, the product phases are not yet evolved. Powder XRDs of early precipitates shows two broad reflections above the amorphous background that somewhat corresponds to the anatase phase. As soon as the product has ample time to crystallize, the amorphous TiO_2_/anatase reflections disappear and sharp rutile reflections peak sharply above the background (*see* Supporting Information, Figure S2). Thus, final products obtained under our synthesis conditions are well crystalline, exhibiting sharp reflections that correspond to the rutile phase of TiO_2_.

To identify crystal formation mechanism the products were studied by TEM. The finest fraction of the precipitate is composed of ultrafine rutile fibers that are commonly aligned into fan-shaped bundles. Alignment of rutile fibers is observed already in the sample processed for only 30 min at the target temperature of 180 °C (Fig. 3a). The length of the rutile fibers can reach up to 500 nm, while their width is just 4–7 nm. After short times of HT treatment a large part of Ti-precursor is yet unreacted (Supporting Information, Figure S2). Consequently, we find numerous isolated rutile fibers embedded in an amorphous gel-like Ti-precursor phase (inset in Fig. 3a; further details in Supporting Information, Figure S3). Due to their high aspect ratio, it is not unusual to see some fibers bent. As may be expected, electron diffraction pattern (EDP) recorded over a rutile bundle shown in Fig. 3a, confirms that the long axis of the fibers corresponds to the c-axis of rutile (Fig. 3b). The most interesting feature is the angular dispersion of {002}_R_ reflections that is caused by systematic misalignment of fibers. Misalignment increases monotonously from the center to the rim of a bundle, in 〈*hk*0〉 directions, producing fan-shaped assembly of fibers. Weak diffraction rings accompanying the strong reflections in EDP correspond to other randomly oriented nanoparticles in the area. While the majority of these rings stem from rutile there is a ring of weak reflections that best matches {101}_A_ of anatase (*see* Fig. 3b; the ring with a slightly larger d-value than {110}_R_ ring of rutile). These weak reflections correspond to clusters of nanosized crystallites with isometric appearance (marked with circle in Fig. 3a), indicating that anatase phase can be present during the initial stages of rutile precipitation. Energy dispersive X-ray spectroscopy (EDS) analysis over the anatase particles or rutile fibers shows no presence of chlorine (from HCl). The 30 min sample was very sensitive to the electron beam, leading to fast irradiation damage.

After 60 min of HT processing at the target temperature of 180 °C, the bundles are generally larger and the rutile fibers are more firmly attached (Fig. 3c). They have poorly defined jagged terminations reflecting their fibrous texture. New features in this sample are shorter rutile rods attached at characteristic angles with respect to the direction of the c-axis of long fibers in the primary bundle. These attachments, reminiscent of twinning, occur at different depths in a bundle. While strong reflections in EDP (Fig. 3d) originate from the bundle, weak diffraction rings stem from the particles that are not aligned with the main bundle. Misalignment of fibers composing the bundle is reflected in an angular spread of reflections containing a component of the rutile c-axis, *e.g.* {101}_R_ and {002}_R_. In this sample anatase particles are no longer present.

### Assembly of rutile fibers

Rutile-type TiO_2_ precipitates in form of few nanometers thick fibers produce fan-shaped nanocrystal assemblies. While initially the fibers are poorly attached and their assembly appears to be weak, this is improved with time, resulting in compacting the bundles, as shown in Fig. 4. Lattice image in Fig. 4a presents the outer section of a bundle from the sample HT treated for 120 min at 180 °C. In the interior, rutile fibers are assembled with such a perfection that they produce nearly ideal crystal lattice, whereas on their surface the crystals are covered by ~1 nm thick envelope of amorphous titania. Bulk rutile is intermittently disrupted with {110} planar faults that suggest assembly of fibers along these low energy prism planes^51^. The distance between the faults corresponds to the average width of precipitated rutile fibers. Any mismatch is compensated by edge dislocations, accompanied with accumulated lattice strain. Near terminations, the fibers can be deflected from the main crystal for several degrees (up to ~7°), embedding amorphous remnants of mineralizing solutions. Facing (110) surfaces are atomically sharp, and at first glance, this might imply splitting and detachment of fibers^42^. However, at the junction between the parent rutile and the fiber, we can observe a formation of additional rutile layers that should not be present, if the crystal was split. A similar ledge growth of rutile can be also observed at outer ends of the parent rutile and the deflected fiber (indicated by circles in Fig. 4a), suggesting a late stage of crystallization rather than crystal splitting. A further evidence for mesocrystal assembly is the presence of amorphous titania enclosed in pores between the imperfectly assembled rutile fibers. A close-up of the interspace in Fig. 4b shows that amorphous material is strongly modulated by the periodicity of adjacent crystalline rutile. Averaged intensity profile across the pocket of amorphous phase shows that its periodicity does not strictly follow the (110) interplanar spacings. The observed modulation can not be a result of contrast delocalization (its effects are seen at the outer amorphous edge) nor they are wedge (same periodicity) or ledge effect that are distinctly different (outlined by circles in Fig. 4a). What we observe here appears to be ordering of mineralizing fluids, frozen in their pristine condition. This evidence suggests that during growth, crystalline matter induces some harmonic ordering that extends 1–2 nm deep into the amorphous phase.

### Multilevel branching of rutile

In addition to lateral assembly of rutile fibers, multiple branching is observed under low supersaturation conditions in highly acidic medium^2^, producing complex crystallographically interconnected 3D architectures. Figure 5 shows a typical branched cluster of rutile crystals. Regular incidences of the branches with respect to the primary crystal are indicative of well-defined crystallographic relation, such as twinning. To confirm twinning crystallographically, the crystals must be oriented into a zone axes, where the twin plane can be viewed edge-on; to view (101) twin in rutile such projections are [010], 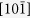, 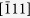, *etc*.^47^ The primary crystal in Fig. 5a is oriented in 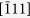 projection, which happens to be the most common docking of branched rutiles on carbon-coated TEM grids. Because in this projection the angle between the c-axes of a branch and the primary crystal cannot be measured directly, we can use prism planes that contain the 〈00*l*〉 vectors. The angle of ~45° measured from the TEM image corresponds to a complementary angle between (110)_R1_ ∢ (110)_R2_ ≡ 134.9°, which is produced by 180° rotation around the (101)-plane normal. This operation generates (101) twin with the characteristic angle between the c-axis of 114.4°, and complementary at 65.6° 18,19,20,45,47. EDPs recorded on the primary crystal and the branch, shown in Fig. 5c,d, confirm this crystallographic relation. On some branches we also observe a 2^nd^ generation of branching (detail shown in Fig. 5b) that is clearly following the same crystallographic rules, as demonstrated by corresponding EDPs in Fig. 5c–f. All rutile crystals displayed in Fig. 5b (R_1_|R_2_, R_2_|R_3_ and R_2_|R_4_) are related by {101} twin relations. Small angular discrepancies are due to imperfect fiber attachment. Nanocrystal attachments that were here identified as twinning, form in a similar process as lateral attachment, already in the initial stages of fiber assembly.

**Figure 1:**
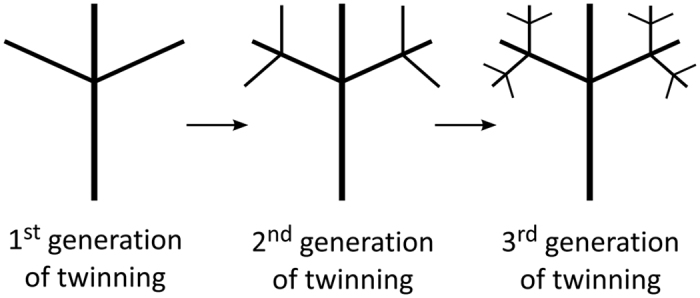
Multilevel branched nanostructures of rutile based on sequential {101} twinning.

**Figure 2:**
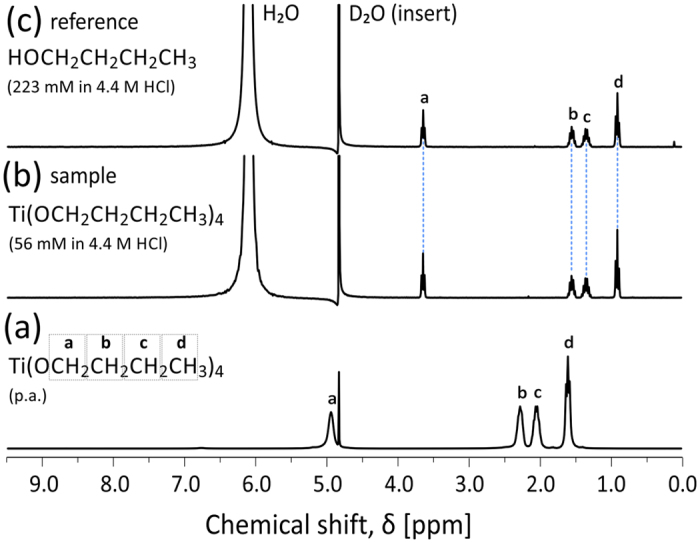
Spectroscopic evidence of RT hydrolysis reaction. ^1^H-NMR spectra of (**a**) 97% Ti(IV)-butoxide (*p.a.*), (**b**) Ti(IV)-butoxide sample and (**c**) n-butanol reference (calculated to match the concentration of n-butanol that is released in the solution after complete hydrolysis).

In rutile we identified two types of oriented attachment: (*i*) lateral assembly of rutile fibers on {110} planes, and (*ii*) twin assembly of rutile fibers on {101} planes, both related to crystallographic planes of the lowest surface energy^51^. Compared to lateral assembly, twin incidences are subordinated by several orders of magnitude. Through fine tuning the HT conditions, *i.e.* precursor and reactant concentrations, acidity, synthesis temperature and time, it is possible to control the equilibrium shape of precipitated crystals and their assembly rate (see Supporting Information; Figure S1). Figure 6 shows the product morphology as a function of precursor concentration, where fan-shaped crystals are formed under high supersaturation conditions, whereas well defined branched rutile structures are produced under low supersaturation conditions. By exploiting two self-assembly modes together with variation of supersaturation tailoring of multilevel branched structures of rutile, such as illustrated in Fig. 1, should be possible.

## Discussion

Based on our results, a possible mechanism for the formation of branched rutile structures can be discussed. In this section we analyze the key processes that are taking place during the formation of rutile from Ti(IV)-butoxide in acidic conditions: (*i*) nucleation of rutile fibers via hydrolysis and condensation, (*ii*) self-assembly of rutile mesocrystals via fiber attachment and growth, and (*iii*) twinning and formation of multilevel branched structures.

### Nucleation of rutile fibers

In order for any TiO_2_-based compound to be produced, the formation of TiO_6_ octahedra, the basic building blocks of these structures, is necessary. The origin of their formation lies in the electronic structure of titanium. In our precursor, Ti(IV)-butoxide, 4s^2^ and 3d^2^ orbitals of titanium undergo an octahedral d^2^sp^3^ hybridization^52^. In this configuration, all four valence electrons located in equatorial hybrid orbitals are shared by highly electronegative OR^−^ ligands (R = C_4_H_9_)^53^, while the two axial orbitals remain empty. In the presence of water, tetravalent cations hydrolyze spontaneously^54^. With ^1^H-NMR analysis we confirmed that even under highly acidic conditions hydrolysis of the precursor is not slowed down^16^,^55^, but is completed already at the room temperature. During hydrolysis, OR^−^ ligands in Ti(IV)-butoxide are substituted by strongly nucleophilic OH^−^ (hydroxo) groups, which are readily generated through dissociation of H_2_O, whereas leaving OR^−^ ligands are protonated to form ROH (alcohol)^53^,^56^. Having 6 bonding orbitals available, Ti^4+^ is prone to form octahedral complexes with ligands^52^. With equatorial orbitals occupied by strongly nucleophilic OH^−^ ligands, the two axial orbitals remain available as acceptors for bonding weak nucleophiles, such as OH_2_ (aquo) in aqueous solutions. Water molecules donate their electron pair towards the empty orbitals of fully positively charged Ti^4+^, whereby its coordination number is increased from four to six, forming a neutral hydroxo-aquo [Ti(OH)_4_(OH_2_)_2_]^0^ complex. Under supersaturation conditions, neutral complexes are readily attracted by van der Waals forces, and with no repulsion forces involved, they are unstable and aggregate in form of amorphous titania^53^,^54^,^56^. In order to form crystalline products, spontaneous condensation must be avoided. This is accomplished by increasing the acidity of reactant medium^50^,^55^. The acid has a dual role; it reduces the initial concentration of dissociated hydroxyl groups *c*[OH^−^] in the solution (*see* Supporting Information; Table S1) and protonates OH^−^ ligands in Ti-hydroxo complex, which increases electrostatic repulsion between the complexes and prevents their immediate condensation^50^,^53^. The degree of protonation is governed by the shift of the hydrolysis equilibrium and increases with acidity forming series of positively charged protonated [Ti(OH)_4−n_(OH_2_)_2+n_]^n+^ |_n=1,2,3_ complexes^54^,^57^.

Positively charged complexes are repelled from each other and they are stable in suspension until the temperature is increased^58^. With thermal agitation, the complexes acquire kinetic energy that is necessary to overcome electrostatic repulsion forces and facilitate their aggregation via condensation reactions that bind hydroxylated assemblies into oxide structures through the removal of H_2_O. In the first step aquo ligands are removed by olation process, producing chains of octahedra. This is followed by oxolation, that replaces the remaining hydroxyl ligands by oxo-bridges and combines the chains into a 3D crystal structure^50^,^54^. The acidity of the medium thus controls the magnitude of repulsion forces through protonation of Ti-complexes; if complexes with higher positive charge are involved in condensation, they will be spontaneously repelled into more stable linear edge-shared configuration, characteristic of rutile^53^. Because of strong repulsion between the protonated complexes, angular configurations, such as characteristic for anatase structure are thus not stable under highly acidic conditions. The oxolation reaction combines linear chains of Ti-octahedra into 3D rutile fibers that gradually condensate from the precursor gel (*see* Supporting Information; Figure S3). During condensation, the positive charge of the polycondensate gradually increases with the length of the chains, which prevents further condensation on the account of increasing partial charge of hydroxo ligands (δ_OH−_), which loose their nucleophilic power (*see* Supporting Information; Table S2)^50^,^54^. At this point, condensation would therefore be stopped unless aquo ligands are deprotonated.

For deprotonation to take place we need highly electronegative species that strongly attract protons. In our system, such species are Cl^−^ ions that are abundantly present in the solution through dissociation of HCl^59^. In polar protic solvents like water, chlorine ions are preferably protonated, rather than coordinating highly charged cation^54^. For this reason it does not appear likely for them to conform a coordination polyhedron with Ti^4+^. Our EDS analysis showed that chlorine is not incorporated into the Ti-polycondensate structures, which indicates that Cl^−^ ions do not bond to Ti-cation, but they may play some indirect role in condensation. Because of the highly positive charge of protonated polycondensates, Cl^−^ ions would be drawn towards the positively charged OH_2_ ligands (*see* Supporting Information; Table S2). Deprotonation events most probably take place during intramolecular proton transfer^57^, when Cl^−^ ions can trap the migrating protons. This reduces the increasingly positive charge of the polycondensate and allows further associative reactions and removal of OH^−^ ligands. In this way, HCl plays the role of catalyst where it: (*i*) enters the process through protonation of complexes during the hydrolysis of Ti(IV)-butoxide (δ_OH−_ increases due to protonation), and (*ii*) departs the process via Cl^−^ assisted deprotonation of highly charged polycondensates (δ_OH−_ approaches an asymptotic level where further condensation is sustained). The catalytic function of HCl is illustrated in Fig. 7.

It is reasonable to assume that starting from their nucleation, the chains would propagate in both directions. As a result, in the central part of the polycondensate oxolation can already start, while on its terminations, olation is still under way. Ratio between the olation and oxolation reactions defines the length of the rutile fibers – under highly acidic conditions long chains of highly protonated Ti-complexes are formed and oxolation is slowed down due to a high number of OH_2_ ligands on their surface and as a result long rutile fibers are formed, on the other hand, if the chains are less protonated, oxolation occurs sooner leading to formation of rutile with rod-like morphology. Similar observations were reported by many researchers^16^,^17^,^29^,^36^. The acidity therefore does not only decelerate condensation^50^, but also determines the balance between the two associative processes, *i.e.* olation and oxolation reactions that control the equilibrium shape of precipitates^36^,^51^.

### Fiber assembly

From the point, when the precipitates reach their equilibrium size they become basic building blocks for assembly of macroscopic mesocrystals through attractive van der Waals forces^3^,^4^. Compared to olation and oxolation reactions, that produce structurally flawless nanocrystals, this associative process is far less perfect (*see* Fig. 4). Although assembled in almost perfect orientation, the interfaces between the constituent blocks are generally incoherent. The main cause of imperfections lies in the fact that their assembly is not driven by chemical (atomic), but rather physical (bulk) interactions. These interactions depend on crystal size, habit, intrinsic electrostatic and magnetic fields, polarization, capping ligands, solvents, particle agitation, *etc*., and their cumulative effect that leads to oriented attachment remains largely unexplored^8^,^49^. Given the complexity of interactions it is astonishing how elementary particles aggregated in mesocrystal assemblies so closely mimic the structure of single crystals^4^,^5^,^13^.

What are the forces that align the nanoparticles? While many authors suggest that either dipolar interactions or intrinsic electric fields are responsible for oriented attachment^3^,^4^,^11^,^12^,^60^, the most recent review on self-assembly of mesocrystals concludes that the nature of these forces is still unknown^49^. Using molecular-dynamics (MD) simulations, Fichthorn^61^ demonstrated that in vacuum anatase crystals are aligned in accordance to their electric fields rather than due to dipolar interactions, whereas in aqueous medium interactions become more complex. Due to repulsion forces of capping ligands on their surface, nanoparticles were found to explore many possible configurations within the interacting electric fields, before they are finally attached. In this process, many transient bonds are formed and broken, if configuration is energetically unstable. When the crystals are finally met in low energy configuration, they are further attracted by interacting crystal fields and fastened in position by hydrogen bonds. In this state, dehydration reactions take place and more stable oxygen bonds are formed in a zipper-like mechanism that closes down the interparticle gap and fixes the crystals in particular orientation. This theoretical study is in many ways consistent with our experimental observations. In our study we provide an experimental evidence of the existence of such envelope of ligands and mineralizing complexes that assist crystallization of nanoparticles through condensation reactions in form of ~1 nm thick amorphous layers surrounding the rutile fibers (Fig. 4). Their width is comparable to adsorption thickness of solvent molecules reported for rutile based on MD simulations and x-ray photoelectron spectroscopy^62^,^63^. High sensitivity to electron irradiation suggests that the surface structure of mesocrystalline rutile is electronically unstable due to unsatisfied bonds^10^. In addition to surface amorphous layers we find, enclosed by imperfectly assembled rutile fibers, pockets of amorphous material that are indicative for mesocrystal formation^3^,^4^. Within these pockets complexes in loosely associated state are organized in semi-periodic manner. Although not forming a discrete structure yet, building blocks (*i.e.* complexes with ligands) show harmonic order that appears to be induced by interplanar spacing of the constraining lattices. The existence of force fields that assist crystallization in a way that building blocks enter the crystal field in an organized manner, before forming chemical bonds, has already been documented in literature. For example, when studying the interface between Al_2_O_3_ and liquid aluminum, Oh *et al*.^64^ observed an interesting phenomenon of ordering in amorphous Al induced by the parent crystal structure. They showed that this ordering propagates up to 6 monolayers into the liquid, which is a similar extent as that observed in our study (Fig. 4). In our case the amorphous material is not a liquid, but a gel-like assembly of charged complexes^65^, which are organized following the underlying crystal structure. The observed harmonic ordering of amorphous material may reflect a frozen state of yet unexplained force fields^49^, that may be the key process of conveying the essential structural information between the particles across a thick protective cloud of ligands and poorly ordered unreacted complexes.

**Figure 3:**
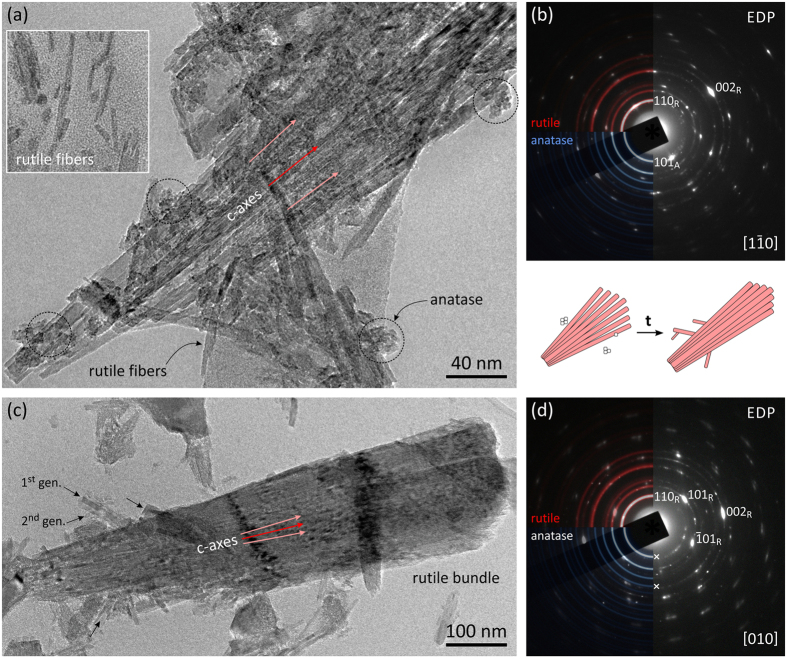
Self-assembly of rutile fibers. TEM of fibrous TiO_2_ precipitates using 56 mM Ti(IV)-butoxide in 4.4 M HCl and a target temperature of 180 °C. (**a**) Already after 30 min the sample contains rutile fibers, roughly aligned along the crystallographic c-axis into fan-shaped assemblies. The inset (left) shows individual rutile fibers precipitated from the amorphous Ti-precursor gel. (**b**) Strong reflections in EDP stem from the assembly of rutile fibers while the rings belong to isolated rutile rods and clusters of nanosized anatase. (**c**) After 60 min, bundles of rutile fibers become more compact and younger generations of rutile with special angular relationships to the primary bundle start to appear. Anatase particles are no longer present; (**d**) in EDP, all reflections belong to rutile. Illustrations show TiO_2_ products developed in the initial stages of processing.

**Figure 4:**
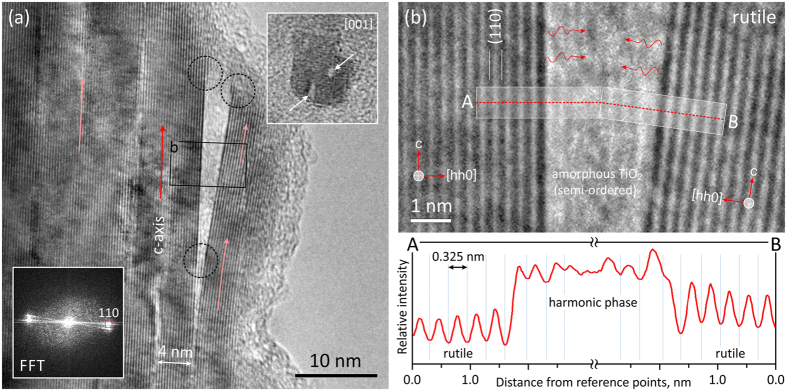
Oriented attachment of rutile fibers. (**a**) High-resolution TEM (HRTEM) image of an edge of a rutile bundle, treated 120 min at 180 °C, using 56 mM Ti(IV)-butoxide in 4.4 M HCl. In bulk rutile, many planar defects are seen along the (110) planes (Fast-Fourier Transform; FFT). The directions of the c-axes are marked by red arrows. The outer stripe of rutile is deflected from the matrix enclosing a pocket of amorphous titania. Dotted circles denote points of secondary crystallization. The inset in the upper-right corner shows a cluster of rutile fibers viewed along the c-axis. White arrows indicate the pores that remained after imperfect assembly of the fibers. (**b**) Close-up of amorphous titania enclosed between two rutile fibers from (**a**). Intensity profile over a phase-contrast image from reference points A–B shows harmonic ordering in the amorphous titania that mimics the (hh0) lattice periodicity of rutile.

When the particles are in movement (by thermal agitation) their electromagnetic fields are coupled^54^, and the particles are aligned parallel to their long axis. Due to multiplicity of interactions the particles are attracted into a loosely associated state^61^,^66^. As the distance between the fibers becomes shorter, by attraction, crystallographic alignment along the lowest energy {110} planes becomes dominant^51^. Oriented assembly of rutile fibers into bundles is observed already in early stages of synthesis (Fig. 3a). With time, the gap between the fibers is closing down (Fig. 3c) until the fibers are packed in highly oriented manner. Elimination of ligands is the last of all processes that finally reduces the overall energy between the two particles^4^,^9^,^10^. In this process, the rutile fibers are merged into a nearly perfect crystal structure, with occasional faults running parallel to {110} planes that witness of the existence of former fibers (Fig. 4). As the nanocrystal assemblies grow larger, further attachments to the existing bundles occur at faster rate. This reduces the chances of perfect alignment with the rest of the bundle. Through continuous condensation of ligands captured in the interparticle space the particles are fixed in accordance to its intrinsic electric field^11^. As a consequence, crystals acquire twisted morphology, which still follows the symmetry rules of the crystal structure but with angular dispersion (*see* illustrations in Fig. 6; and Supporting Information, Figure S1). This kind of twisting appears to be the main macroscopic evidence of mesocrystal assembly^3^,^4^. The described mechanism of (110) lateral attachment of rutile fibers is illustrated in Fig. 8.

### Crystal branching (twinning)

In literature, twinning in rutile has been attributed to either oriented attachment^18^,^19^,^40^,^49^, topotaxial replacement reactions^46^,^47^,^48^, or deformation^47^. To produce twins by mechanical deformation rutile crystals must be subjected to a considerable compressive stress of >100 MN/m^2^ at elevated temperatures (>400 °C) that generates the necessary energy to activate slip systems to move dislocations^67^. Under HT conditions, where the crystals grow unrestrained, deformation-induced twinning can thus be excluded. For replacement reaction to take place we would need a crystalline precursor phase with structurally related sublattice that is transformed into rutile^46^,^47^,^48^. Indeed, under some synthesis conditions, the twins have a single nucleation point, suggesting that they form during nucleation of rutile^40^,^43^,^44^,^45^. Under our experimental conditions the first precipitate is fibrous rutile with amorphous titania and minor anatase. While anatase and rutile have no 3D structural relation, they are polymorphs of the same compound and participation of anatase in rutile twinning cannot be entirely ruled out.

We confirmed the presence of transient anatase phase after short synthesis times that disappeared in continued HT treatment. The presence of anatase in the initial stages of rutile precipitation is consistent with observations of other researchers^19^, and can be explained by a rapid condensation and abundant nucleation after short processing times at low temperatures^54^. As soon as the temperature increases or the time of thermal treatment is extended, anatase nuclei dissolve and recrystallize to rutile. Conditions for the formation of anatase are met at the beginning of HT treatment, when the target temperature is not yet achieved and the concentration of complexes is high. Interestingly, anatase nanocrystals are clustering independently of rutile into their own mesocrystalline assemblies^68^, indicating that a similar physically driven oriented attachment mechanism controls assembly of anatase nanoparticles, as described above for rutile. At elevated temperatures, where supersaturation is lower^2^, and thermally driven kinetic energy of the particles is larger, anatase becomes unstable and disintegrates^54^. In HT medium, anatase probably does not disintegrate into simple ions that would take part in coarsening of rutile through Ostwald’s ripening mechanism but is rather protonated (repulsion!) to form elementary building blocks that then, assisted by thermal agitation, rearrange into rutile. Based on the presence of this transient anatase phase, Li *et al*.^19^ inferred that twinning of rutile is mediated by anatase-to-rutile transformation. In our TEM investigation we could not confirm any epitaxial relationship between the two polymorphs under employed HT conditions.

**Figure 5:**
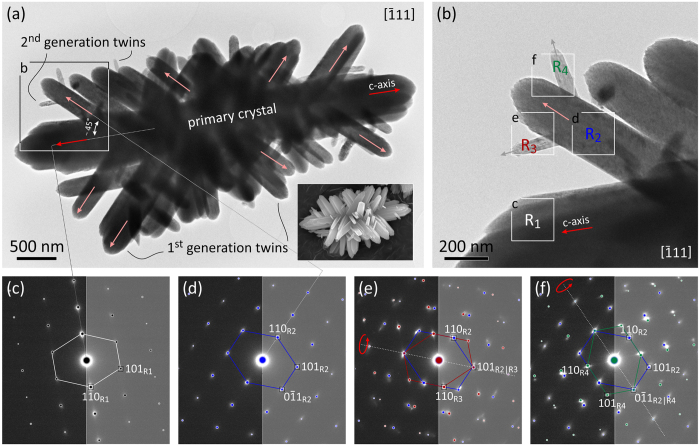
TEM study of twinning in multilevel branched rutile cluster. (**a**) Branched rutile cluster in 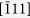 projection with discernable crystallographic incidences. The inset shows an SEM image of branched rutile cluster. Typical angle between the primary crystal and the branches, measured between (110) planes is ~45°. Dotted lines drawn to corresponding EDPs (**c**,**d**) assist the measurement. EDP in (**d**) is related to EDP in (**c**) by 180° rotation around the (101)-plane normal, which corresponds to (101) twin operation. The inset shows an SEM image of a similar twin cluster. (**b**) Second generation of branching follows the same crystallographic rules. Squares indicate the locations of selected area EDPs in (**c**–**f**). Colored dots represent simulated diffraction patterns for: R_1_–primary crystal, R_2_–1^st^ generation twin, R_3_ and R_4_–2^nd^ generation twins. Small angular dispersion of diffraction spots stems from lateral dispersion of c-axes caused by imperfect fiber attachment. The sample was synthesized using 56 mM Ti(IV)-butoxide in 4.4 M HCl and HT treated at 180 °C for 1 hour.

**Figure 6:**
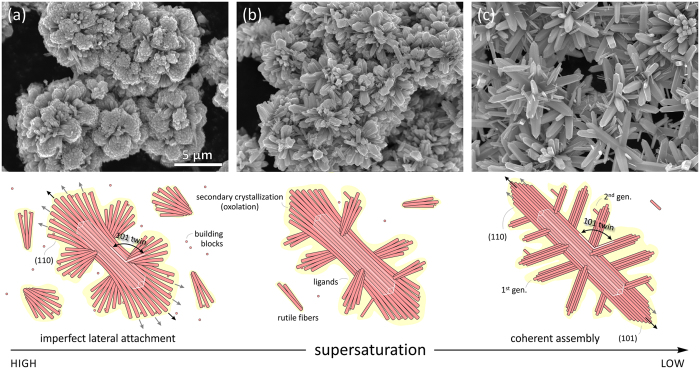
The effect of precursor concentration of the morphology of the product. (**a**) Under high supersaturation conditions (*i.e.* high precursor concentration; 83 mM of Ti(IV)-butoxide), bundles of radiating rutile crystals are formed, producing spherulitic particles. Each bundle is composed of minute rutile crystals twisted few degrees away from the common c-axis, whereas individual bundles in such a cluster appear to be linked by twinning. (**b**) With decreasing supersaturation (56 mM of Ti(IV)-butoxide) the alignment between the rutile becomes more perfect while their length increases. (**c**) On even further decrease of supersaturation (28 mM of Ti(IV)-butoxide) well-defined multilevel branched crystals of rutile are assembled through perfect lateral assembly and twinning. The samples were synthesized using 4.4 M HCl and HT treated at 180 °C for 3 hours. Illustrations show rutile clusters developed at various degrees of supersaturation.

**Figure 7:**
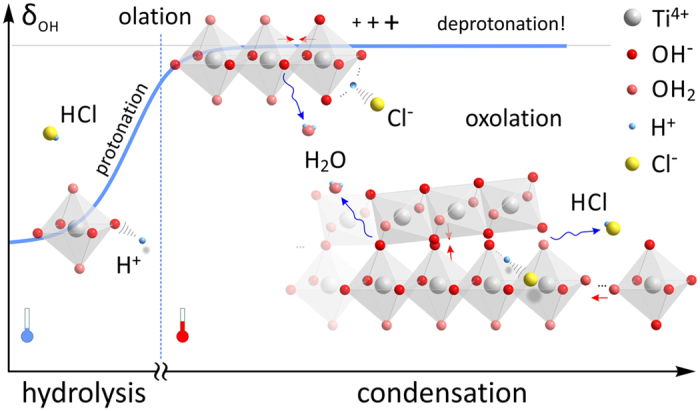
Catalytic role of HCl during hydrolysis and condensation processes. In acidic medium, HCl protonates OH^−^ ligands in Ti(IV)-complexes, making them stable at ambient conditions. At elevated temperatures the hydroxo-aquo complexes loose water through associative nucleophilic reactions to form chains of Ti-octahedra (olation). With their length charge increases to the point when free Cl^−^ ions can trap migrating protons and deprotonate growing polycondensate, allowing further release of water (oxolation) and their assembly into the rutile structure.

**Figure 8:**
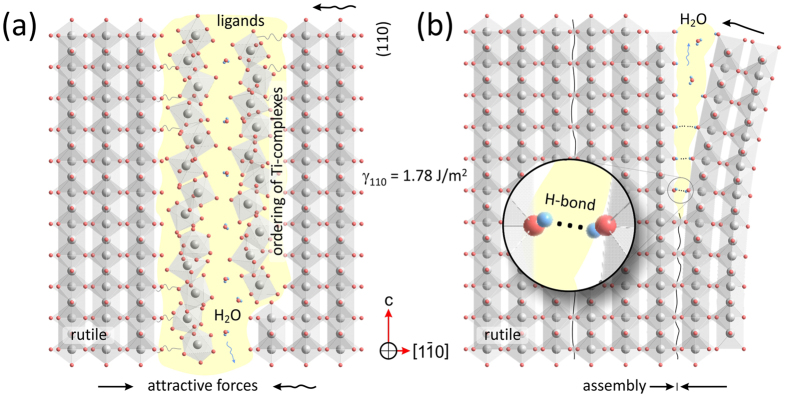
Illustration of lateral fiber assembly along {110} planes of rutile. (**a**) Precipitated rutile fibers are brought into near-crystallographic orientation by the long-range electromagnetic force field that conveys the essential structural information across a thick protective cloud of poorly ordered ligands and unreacted complexes. The force-field induces harmonic ordering of complexes before they are built into the crystal structure. Ligands and complexes adsorbed on the surface of nanoparticles prevent immediate aggregation, allowing the nanoparticles to explore many possible configurations within the interacting electric fields before they are finally attached. When the distance between the fibers becomes shorter, crystallographic alignment along the lowest energy {110} planes (Oliver *et al*.)^51^ becomes dominant. This is followed by elimination of ligands, which finally reduces the overall energy between the two particles. (**b**) Particles are fastened in a position by hydrogen bonds followed by the dehydration reactions, where more stable oxygen bonds are formed in a zipper-like mechanism that closes down the interparticle gap and fixes the crystals in lateral orientation.

**Figure 9:**
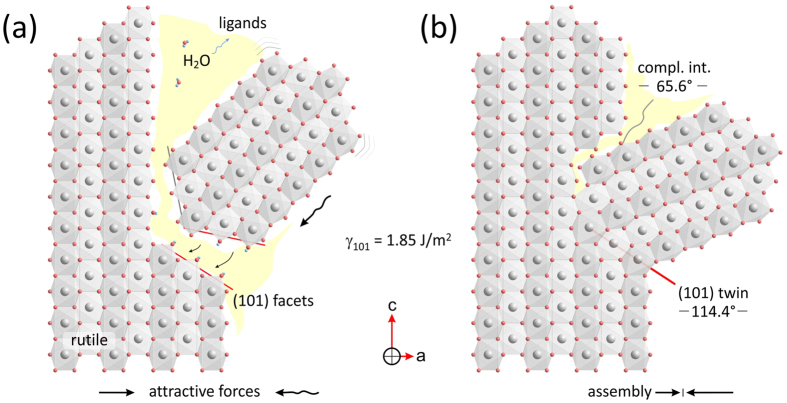
Illustration of twin assembly along {101} planes of rutile. (**a**) After Oliver *et al*.^51^ the {101} are the planes with the second lowest surface energy in rutile. Like {110} planes these can also serve for oriented attachment of suitably oriented rutile fibers. First, the two fibers are brought into near-crystallographic orientation by the attractive electromagnetic forces. This process is likely to take place on surface steps or terminations, where (101) facets are exposed. Unlike lateral attachment this assembly mode brings the two crystals into a (101) twin orientation, that represents one of the lowest energy configurations for rutile. (**b**) This is followed by the ligand elimination via dehydration reactions, which fixes the fibers in (101) twin orientation with characteristic angle of 114.4° between the c-axis, and its complementary at 65.6°.

Under our synthesis conditions, twinning can occur at any time during growth of rutile, producing branched structures (Fig. 5). As the twinned crystals are not in exact crystallographic relation with respect to the general orientation of the primary crystal (*see* Fig. 6), their origin must be related to imperfect oriented attachment^40^,^49^. Our observations imply that lower is the surface energy of the specific crystallographic face, higher is the affinity for its oriented attachment. Based on calculated surface energies for rutile by Oliver *et al*.^51^, {110} faces have the lowest energy, followed by {101} and {001} surfaces. If attachment on {110} planes leads to lateral assembly of rutile crystals, their attachment on {101} planes leads to twinning. Because of slightly larger surface energy and smaller surface area of {101} surfaces, twinning incidences are less frequent than {110} attachment of fibers. They appear to form by oriented attachment of single fibers on {101} steps of rutile bundles at any stage of mesocrystal aggregation. As soon as a fiber is attached in twin orientation, it starts to attract other fibers through lateral attachment. All attachment events are crystallographically imperfect and the interfaces are incoherent. In morphological study of branching in rutile Huang *et al*.^20^, pointed out that twins are not firmly attached (easily broken off) indicating their imperfect attachment. In addition to {101} twins no other type of twinning is present, except for complementary boundaries^48^, that are produced by impingement of rutile domains in {101} twin orientation. Multiple twinning results in tree-like branched morphology. Because twinning occurs throughout the assembly process, we can obtain fractal-like multi-branched structures, with several generations of twinning. The described mechanism of (101) twin attachment of rutile fibers is illustrated in Fig. 9.

In summary, we provide experimental evidence of theoretically predicted electric force fields that control oriented attachment of crystals. We have shown that multilevel twinned TiO_2_ structures are produced by mesocrystal assembly following two sets of low-energy planes {110} and {101} where the degree of branching can be controlled by the choice of experimental conditions. These two self-assembly mechanisms provide the protocol for tailoring desirable 3D branched TiO_2_-based architectures.

## Methods

### Hydrothermal Synthesis

Rutile-type TiO_2_ was synthesized following the procedure reported by Zhou *et al*.^16^ which produces most versatile branched structures. To study the role of precursor concentration and the acidity of the reactant medium on aggregation and branching of crystals, chosen amount of titanium (IV) butoxide (Ti(IV)-butoxide; 97% Sigma-Aldrich) was added dropwise into 4.4 or 5.5 M aqueous solutions of hydrochloric acid (HCl; 37% Sigma-Aldrich) and mixed until the solution was completely clear. Calculated to the total volume, precursor concentrations varied from 83–28 mM. The resulting solution was used for the proton nuclear magnetic resonance (^1^H-NMR) analysis. Additionally, for the unambiguous confirmation of our results we prepared a mixture of n-butanol (99% Alfa Aesar) in 4.4 HCl following the same procedure. The concentration of n-butanol was set to 223 mM, which corresponds to the concentration of n-butanol released in the solution after complete hydrolysis of 56 mM Ti(IV)-butoxide. For hydrothermal (HT) treatment, 2/5 of 23 ml Teflon-lined stainless steel autoclave (Model 4749, Parr Instrument Company) was filled with the resulting solution and heated at different target temperatures (140–180 °C) and times (0.5–10 h). In our experiments the autoclaves with reagent mixtures were put into the preheated oven. After HT processing the autoclave was cooled to a room temperature and white precipitate from the solution was collected, centrifuged, washed in an absolute ethanol and dried in air. Under extremely acidic conditions and short synthesis times, where the amount of obtained precipitate was low, HCl was first evaporated and then the product was washed in absolute ethanol and dried.

### Materials Characterization

Analysis of the starting solution before HT treatment was performed with ^1^H-NMR (300 MHz Agilent-Varian) spectrometer, with the use of 5 mm Wilmad^®^ NMR sample tubes. Chemical shifts (δ) are reported with respect to TMS. Deuterium oxide (D_2_O; 100 at% Armar Chemicals) in coaxial inset was used as external lock. Phase composition and crystallinity of HT grown products were analyzed by powder X-ray diffraction (XRD) analysis (PANalyical X’Pert High-Resolution PRO diffractometer). The morphology of synthesized branched TiO_2_ crystals was subsequently studied using a field-emission scanning electron microscope (FEG-SEM; model Supra^TM^ 3VP, Carl Zeiss). Based on their morphological features samples were selected for further studies of aggregation and twinning by transmission electron microscopy (TEM; model JEM 2100, Jeol Ltd., Tokyo, Japan), employing selected area electron diffraction (SAED) and high-resolution (HRTEM) techniques that enable an insight into the fine structural features of branched rutile crystals. The samples for TEM studies were prepared by suspending the product in an absolute ethanol and depositing a drop of the fine fraction on lacey carbon coated TEM grids and dried in air before observation.

## Additional Information

**How to cite this article**: Jordan, V. *et al*. Self-assembly of multilevel branched rutile-type TiO_2_ structures via oriented lateral and twin attachment. *Sci. Rep.* **6**, 24216; doi: 10.1038/srep24216 (2016).

## Supplementary Material

Supplementary Information

## References

[b1] Cheng C. & Fan H. J. Branched nanowires: Synthesis and energy applications. Nano Today 7, 327–343 (2012).

[b2] Cao G. Nanostructures & Nanomaterials Synthesis, Properties & Applications (Imperial College Press: London UK, 2004).

[b3] Cölfen H. & Antonietti M. Mesocrystals: Inorganic superstructures made by highly parallel crystallization and controlled alignment. Angew. Chem. Int. Ed. 44, 5576–5591 (2005). 10.1002/anie.200500496 16035009

[b4] Niederberger M. & Cölfen H. Oriented attachment and mesocrystals: Non-classical crystallization mechanisms based on nanoparticle assembly. Phys. Chem. Chem. Phys. 8, 3271–3287 (2006). 16835675 10.1039/b604589h

[b5] Žagar K., Rečnik A., Ajayan P. M. & Čeh M. Oriented cube-on-cube nanocrystal assembly of SrTiO_3_. Nanotechnology 21, 375605–375612 (2010). 20720291 10.1088/0957-4484/21/37/375605

[b6] Park J., Privman V. & Matijevič E. Model of formation of monodispersed colloids. J. Phys. Chem. B 105, 11630–11635 (2001).

[b7] Vayssieres L., Beermann N., Lindquist S. E. & Hagfeldt A. Controlled aqueous chemical growth of oriented three-dimensional crystalline nanorod arrays: Application to iron (III) oxides. Chem. Mater. 13, 233–235 (2001).

[b8] Zhang S. Y., Regulacio M. D. & Han M. Y. Self-assembly of colloidal one dimensional nanocrystals. Chem. Soc. Rev. 43, 2301–2323 (2014). 24413386 10.1039/c3cs60397k

[b9] Lee Penn R. Kinetics of oriented aggregation. J. Phys. Chem. B 108, 12707–12712 (2004).

[b10] Lee Penn. R. & Banfield J. F. Imperfect oriented attachment: Dislocation generation in defect-free nanocrystals. Science 281, 969–971 (1998). 9703506 10.1126/science.281.5379.969

[b11] Simon P., Zahn D., Lichte H. & Kniep R. Intrinsic electric dipole fields and the induction of hierarchical form developments in fluorapatite–gelatine nanocomposites: A general principle for morphogenesis of biominerals? Angew. Chem. Int. Ed. 45, 1911–1915 (2006). 10.1002/anie.200504465 16493721

[b12] Kniep R., Simon P. & Rosseeva E. Structural complexity of hexagonal prismatic crystal specimens of fluorapatite-gelatine nanocomposites: A case study in biomimetic crystal research. Cryst. Res. Technol. 49, 4–13 (2014).

[b13] Liu Z. . Intrinsic dipole-field-driven mesoscale crystallization of core-shell ZnO mesocrystal microspheres. J. Am. Chem. Soc. 131, 9405–9412 (2009). 19518047 10.1021/ja9039136

[b14] Chen X. & Mao S. S. Titanium dioxide nanomaterials: Synthesis, properties, modifications, and applications. Chem. Rev. 107, 2891–2959 (2007). 17590053 10.1021/cr0500535

[b15] Nguyen Phan T. D. . A simple hydrothermal preparation of TiO_2_ nanomaterials using concentrated hydrochloric acid. J. Cryst. Growth. 312, 79–85 (2009).

[b16] Zhou W. . Control synthesis of rutile TiO_2_ microspheres, nanoflowers, nanotrees and nanobelts via acid-hydrothermal method and their optical properties. CrystEngComm 13, 4557–4563 (2011).

[b17] Sarkar D., Ghosh C. K. & Chattopadhyay K. K. Morphology control of rutile TiO_2_ hierarchical architectures and their excellent field emission properties. CrystEngComm 14, 2683–2690 (2012).

[b18] Wang H. . Hierarchical rutile TiO_2_ mesocrystals assembled by nanocrystals-oriented attachment mechanism. CrystEngComm 14, 2278–2282 (2012).

[b19] Li D. . Growth mechanism of highly branched titanium dioxide nanowires via oriented attachment. Cryst. Growth Des. 13, 422–428 (2013).

[b20] Huang Y. S. & Liu H. W. Growth morphologies of nanostructured rutile TiO_2_. J. Mater. Eng. Perform. 23, 1240–1246 (2014).

[b21] Mo S. D. & Ching W. Y. Electronic and optical properties of three phases of titanium dioxide: Rutile, anatase, and brookite. Phys. Rev. B 51, 13023–13032 (1995). 10.1103/physrevb.51.13023 9978097

[b22] Linsebigler A. L., Lu G. & Yates J. T. Photocatalysis on TiO_2_ surfaces: Principles, mechanisms, and selected results. Chem. Rev. 95, 735–758 (1995).

[b23] Zhang H. & Banfield J. F. Thermodynamic analysis of phase stability of nanocrystalline titania. J. Mater. Chem. 8, 2073–2076 (1998).

[b24] Hanaor D. A. H. & Sorrell C. C. Review of the anatase to rutile phase transformation. J. Mater. Sci. 46, 855–874 (2011).

[b25] Banerjee A. N. The design, fabrication, and photocatalytic utility of nanostructured semiconductors: focus on TiO_2_-based nanostructures. Nanotechnol. Sci. Appl. 4, 35–65 (2011). 24198485 10.2147/NSA.S9040 PMC3781710

[b26] Scanlon D. O. . Band alignment of rutile and anatase TiO_2_. Nat. Mater. 12, 798–801 (2013). 23832124 10.1038/nmat3697

[b27] Zhu T. & Gao S. P. The stability, electronic structure, and optical property of TiO_2_ polymorphs. J. Phys. Chem. C 118, 11385–11396 (2014).

[b28] Fujishima A., Zhang X. & Tryk D. A. TiO_2_ photocatalysis and related surface phenomena. Surf. Sci. Rep. 63, 515–582 (2008).

[b29] Kobayashi M., Petrykin V. & Kakihana M. Hydrothermal synthesis and photocatalytic activity of whicker-like rutile-type titanium dioxide. J. Am. Ceram. Soc. 92, S21–S26 (2009).

[b30] Cho I. S. . Branched TiO_2_ nanorods for photoelectrochemical hydrogen production. Nano Lett. 11, 4978–4984 (2011). 21999403 10.1021/nl2029392

[b31] Krivec M., Segundo R. A., Faria J. L., Silva A. M. T. & Dražić G. Low-temperature synthesis and characterization of rutile nanoparticles with amorphous surface layer for photocatalytic degradation of caffeine. Appl. Catal. B Environ. 140–141, 9–15 (2013).

[b32] Oh J. K., Lee J. K., Kim H. S., Han S. B. & Park K. W. TiO_2_ branched nanostructure electrodes synthesized by seeding method for dye-sensitized solar cells. Chem. Mater. 22, 1114–1118 (2010).

[b33] Sheng J. . Formation of single-crystalline rutile TiO_2_ splitting microspheres for dye-sensitized solar cells. Sol. Energy 85, 2697–2703 (2011).

[b34] Chen R. & Wang M. Synthesis of hierarchical TiO2 micro/nanostructure and its application in hybrid solar cell. Mater. Lett. 69, 41–44 (2012).

[b35] Ye M., Liu H. Y., Lin C. & Lin Z. Hierarchical rutile TiO_2_ flower cluster-based high efficiency dye-sensitized solar cells via direct hydrothermal growth on conducting substrates. Small 9, 312–321 (2013). 23047462 10.1002/smll.201201590

[b36] Lin J. . 3D hierarchical rutile TiO_2_ and metal-free organic sensitizer for producing dye-sensitized solar cells 8.6% conversion efficiency. Sci. Rep. 4, 1–8 (2014). 10.1038/srep05769 PMC5385835 25167837

[b37] Savage N. . Composite n-p semiconducting titanium oxides as gas sensors. Sens. Actuators, B 79, 17–27 (2001).

[b38] Varghese O. K. . Extreme changes in the electrical resistance of titania nanotubes with hydrogen exposure. Adv. Mater. 15, 624–627 (2003).

[b39] Yang X. . Hierarchically nanostructured rutile arrays: Acid vapor oxidation growth and tunable morphologies. ACS Nano 3, 1212–1218 (2009). 19400581 10.1021/nn900084e

[b40] Tsai M. H., Chen S. Y. & Shen P. Imperfect oriented attachment: Accretion and defect generation of nanosized rutile condensates. Nano Lett. 4, 1197–1201 (2004).

[b41] He M., Yu L., Lu X. & Feng X. Large-scale hydrothermal synthesis of twinned rutile titania. J. Am. Ceram. Soc. 90, 319–321 (2007).

[b42] Cha S. I. . Crystal splitting and enhanced photocatalytic behavior of TiO_2_ rutile nano-belts induced by dislocations. Nanoscale, 5, 753–758 (2013). 23223582 10.1039/c2nr33028h

[b43] Yang K. . Sonochemical synthesis and microstructure investigation of rod-like nanocrystalline rutile titania. Mater. Lett. 57, 4639–4642 (2003).

[b44] Li G. L. & Wang G. H. Morphologies of rutile form TiO_2_ twins crystals. J. Mater. Sci. Lett. 18, 1243–1246 (1999).

[b45] Lu W. . Large scale synthesis of V-shaped rutile twinned nanorods. CrystEngComm 14, 3120–3124 (2012).

[b46] Daneu N., Schmid H., Rečnik A. & Mader W. Atomic structure and formation mechanism of (301) rutile twins from Diamantina (Brazil). Am. Mineral. 92, 1789–1799 (2007).

[b47] Daneu N., Rečnik A. & Mader W. Atomic structure and formation mechanism of (101) rutile twins from Diamantina (Brazil). Am. Mineral. 99, 612–624 (2014).

[b48] Rečnik A., Stanković N. & Daneu N. Topotaxial reactions during the genesis of oriented rutile/hematite intergrowths from Mwinilunga (Zambia). Contrib. Mineral. Petrol. 169, 19 (2015).

[b49] De Yoreo J. J. . Crystallization by particle attachment in synthetic, biogenic, and geologic environments. Science 349, aaa6760, doi: 10.1126/science.aaa6760 (2015). 26228157

[b50] Livage J., Henry M. & Sanchez C. Sol-gel chemistry of transition metal oxides. Progr. Solid St. Chem. 18, 259–341 (1988).

[b51] Oliver P. M., Watson G. W., Kelsey E. T. & Parker S. C. Atomistic simulation of the surface structure of the TiO_2_ polymorphs rutile and anatase. J. Mater. Chem. 7, 563–568 (1997).

[b52] House J. E. & House K. A. Descriptive Inorganic Chemistry. 2^nd^ Ed (Elsevier Inc.: Oxford UK, 2010).

[b53] Gopal M., Moberly Chan W. J. & De Jonghe L. C. Room temperature synthesis of crystalline metal oxides. J. Mater. Sci. 32, 6001–6008 (1997).

[b54] Jolivet J.-P., Henry M. & Livage J. Metal Oxide Chemistry and Synthesis (John Wiley & Sons, Ltd: West Sussex England, 2000).

[b55] Bai X., Xie B., Pan N., Wang X. & Wang H. Novel tree-dimensional dendelon-like TiO_2_ structure with high photocatalytic activity. J. Solid State Chem. 181, 450–456 (2008).

[b56] Buyuktas B. S. Investigation of the complexation and hydrolysis-condensation of titanium (IV)n-butoxide [Ti(Obu^n^)_4_] with some unsaturated mono and dicarboxylic acids. Transit. Metal Chem. 31, 786–791 (2006).

[b57] Henry M., Jolivet J.-P. & Livage J. Aqueous Chemistry of Metal Cations: Hydrolysis, Condensation and Complexation (Springer-Verlag, Berlin Germany, 1992).

[b58] Yanqing Z., Erwei S., Zhizhan C., Wenjun L. & Xingfang H. Influence of solution concentration on the hydrothermal preparation of titania crystallites. J. Mater. Chem. 11, 1547–1551 (2001).

[b59] Lee C., Sosa C., Planas M. & Novoa J. J. A theoretical study of the dissociation of HT, HCl, and H_2_S in water clusters. J. Chem. Phys. 104, 7081–7085 (1996).

[b60] Ghezelbash A., Koo B. & Korgel B. A. Self-assembled stripe patterns of CdS nanorods. Nano Lett. 6, 1832–1836 (2006). 16895382 10.1021/nl061035l

[b61] Fichthorn K. A. Atomic-scale aspects of oriented attachment. Chem. Eng. Sci. 121, 10–15 (2015).

[b62] Předota M. . Electric double layer at the rutile (110) surface. 1. Structure of surfaces and interfacial water from molecular dynamics by use of ab-initio potentials. J. Phys. Chem. B 108, 12049–12060 (2004).

[b63] Ketteler G. . The nature of water nucleation sites on TiO_2_ (110) surfaces revealed by ambient pressure X-ray photoelectron spectroscopy. J. Phys. Chem. C 111, 8278–8282 (2007).

[b64] Oh S. H., Kauffmann Y., Scheu C., Kaplan W. D. & Rühle M. Ordered liquid aluminium at the interface with sapphire, Science 310, 661–663 (2005). 16210498 10.1126/science.1118611

[b65] Ivanov V. K., Fedorov P. P., Ye Baranchikov A. & Osiko V. V. Oriented attachment of particles: 100 years of investigations of non-classical crystal growth. Russ. Chem. Rev. 83, 1204–1222 (2014).

[b66] Li D. . Direction-specific interactions control crystal growth by oriented attachment. Science 336, 1014–1018 (2012). 22628650 10.1126/science.1219643

[b67] Takeuchi S. & Hashimoto T. Deformation mechanisms in titanium dioxide single crystals. J. Mater. Sci. 25, 417–423 (1990).

[b68] Da Silva R. O., Gonçalves R. H., Stroppa D. G., Ramirez A. J. & Leite E. R. Synthesis of recrystallized anatase TiO_2_ mesocrystals with Wulff shape assisted by oriented attachment. Nanoscale 3, 1910–1916 (2011). 21423940 10.1039/c0nr01016b

