# Supplementary material for: Self-assembly of multilevel branched rutile-type TiO2 structures via oriented lateral and twin attachment
Source: Sci Rep. 2016 Apr 11;6:24216. doi: 10.1038/srep24216 (PMC4827082; doi:10.1038/srep24216)
Supplement: Supplementary Information [file srep24216-s1.doc]

Self-assembly of multilevel branched rutile-type TiO_2_
structures via oriented lateral and twin attachment

Vanja Jordan,^1^ Uroš Javornik,^2^ Janez Plavec,^2^ Aleš Podgornik,^3^ and Aleksander Rečnik^1^,*

*Department for Nanostructured Materials, Jožef Stefan Institute & Jožef Stefan International Postgraduate School, Jamova cesta 39, SI-1000 Ljubljana, Slovenia.*

*^2^ Slovenian NMR centre, National Institute of Chemistry, Hajdrihova 19, SI-1000 Ljubljana, Slovenia.*

*^3^ Centre of Excellence for Biosensors, Instrumentation and Process Control, Tovarniška 26, SI-5270 Ajdovščina, Slovenia.*

** the corresponding author email: aleksander.recnik@ijs.si*

The following materials provide additional information on the influence of HT synthesis parameters on the morphology and phase composition of the products, calculations for OH^-^/H^+^ ratio and partial charges (δ) of ligands for polycondensates produced by the olation process.

**Calculation of OH^-^/H^+^ ratio:**

By increasing the acidity of the reactant medium, OH^-^/H^+^ ratio is decreased, *i.e.* c[OH^-^] is reduced. For the calculation of OH^-^/H^+^ ratio we can use two equations:^1^

K_H2O_ = c[H_3_O^+^] ∙ c[OH^-^] = 10^-14^ (1)

c[OH^-^] = *K*_H2O_/c[H^+^] (2)

where *K*_H2O_ is the dissociation constant of water and c[H^+^] is the molar concentration of the acid. Reduction of c[OH^-^] and OH^-^/H^+^ ratio when using hydrochloric acid are summarized in Table S1.

**Table S1 |** Calculated values for c[OH^-^] and OH^-^/H^+^ ratio for pure water and hydrochloric acid of different molarities, showing their decrease as a function of acid concentration.

| **c[OH^-^] [M]** | | **OH^-^/H^+^ ratio [/]** | |  |
| --- | --- | --- | --- | --- |
| H_2_O | 10^-7^ | | 1 | |
| 4.4 M HCl | 2.27∙10^-15^ | | 5.16∙10^-16^ | |
| 5.5 M HCl | 1.82∙10^-15^ | | 3.31∙10^-16^ | |


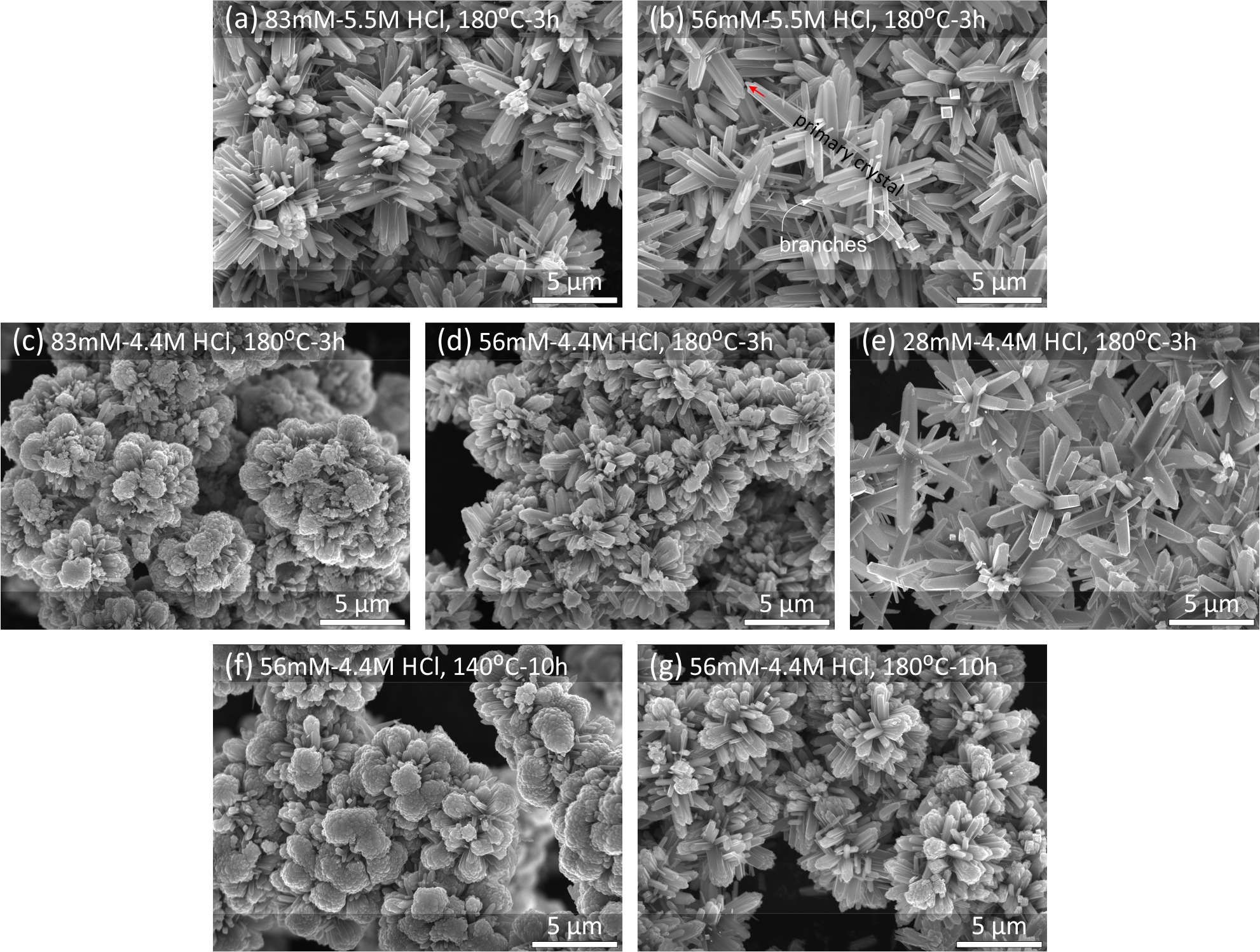


**Figure S1 |Reactive HT processing of Ti(IV)-butoxide showing different modes of branching under varied synthesis conditions**. HT synthesis using 5.5 M HCl (low reactant conditions, *i.e.* low OH^-^/H^+^ ratio) with Ti(IV)-butoxide precursor concentrations of (**a**) 83 mM and (**b**) 56 mM and target temperature of 180 °C for 3 h, yields longer rutile crystals which are displaying similar angular contacts as those obtained at higher reactant and lower precursor concentrations, *i.e.* (d) and (e). The main difference between (a) and (b) is noticeable by the thickness of primary crystals; at higher precursor concentration (83 mM) primary crystals are thicker when compared to the primary crystals obtained at lower precursor concentration (56 mM). Images (**c**) – (**e**) show products obtained at higher reactant concentrations (4.4 M HCl) as a function of decreasing precursor concentration at constant temperature and time (180 °C and 3 h). (**c**) With higher precursor concentration, spherulitic particles consisted of numerous ultrafine rutile fibers radiating from the common center are formed. (**d**) With decreasing concentration of precursor the rutile particles are less dense and show fewer and more visible branches where numerous possible twin incidences and other types of intergrowths can be identified. (**e**) On further decrease of precursor concentration well defined multilevel branched crystals of rutile are produced. (**f**) At lower temperature (140 °C) the products become spherulitic, indicating higher degree of supersaturation than that at higher temperature, *i.e.* nuclei are smaller and consequently their concentration is higher.^2^ (**g**) The increase of synthesis time (10 h) shows no significant influence on the final product’s morphology, compared with products in (d) and (g). In summary, for production of highly branched rutile-type TiO_2_ (based on twinning) we need the following HT conditions: (*i*) high temperature, (*ii*) low precursor or (*iii*) low reagent concentration.


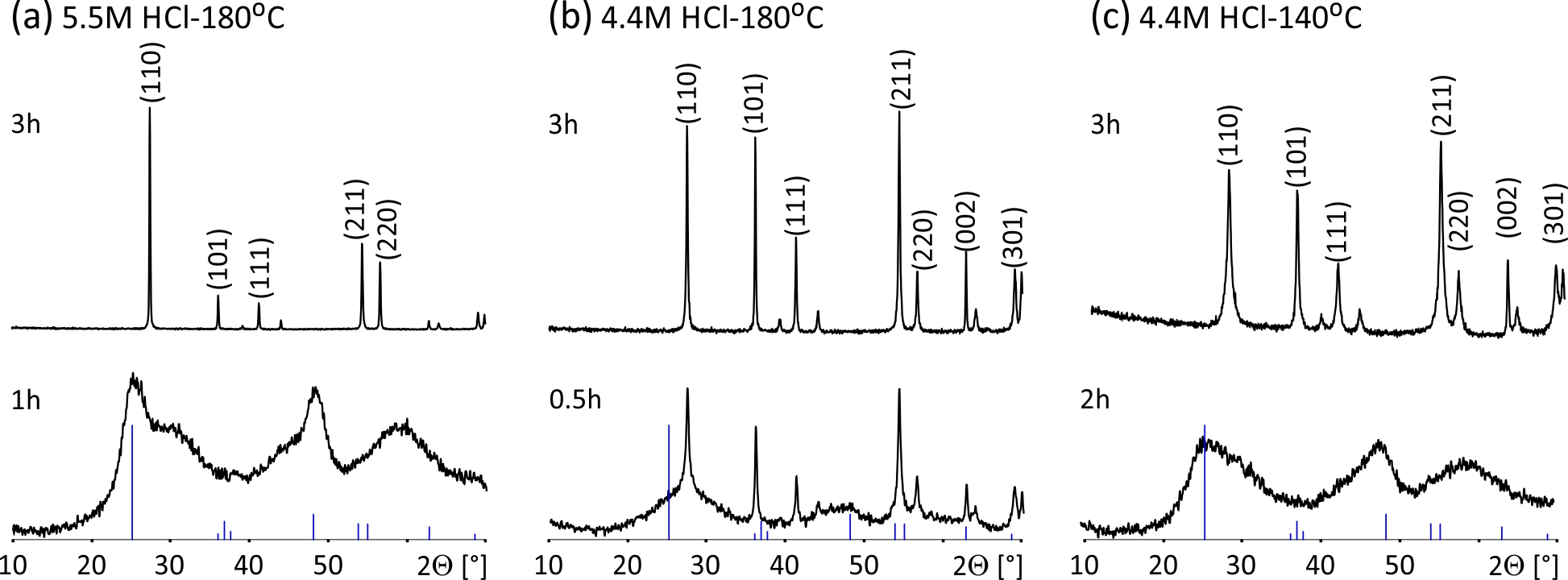


**Figure S2 |** **XRD patterns of HT synthesized rutile**: (**a**) in 5.5 M HCl at target temperature of 180 °C for 1 and 3 h, (**b**) in 4.4 M HCl at target temperature of 180 °C for 0.5 and 3 h, and (**c**) in 4.4 M HCl at target temperature of 140 °C for 2 and 3 h. Ti(IV)-butoxide concentration was held at 56 mM. For all compositions sharp reflections correspond to the rutile-type TiO_2_. After shorter processing times (below) these compositions show the presence of amorphous material, suggesting anatase-type TiO_2_ ordering (blue).


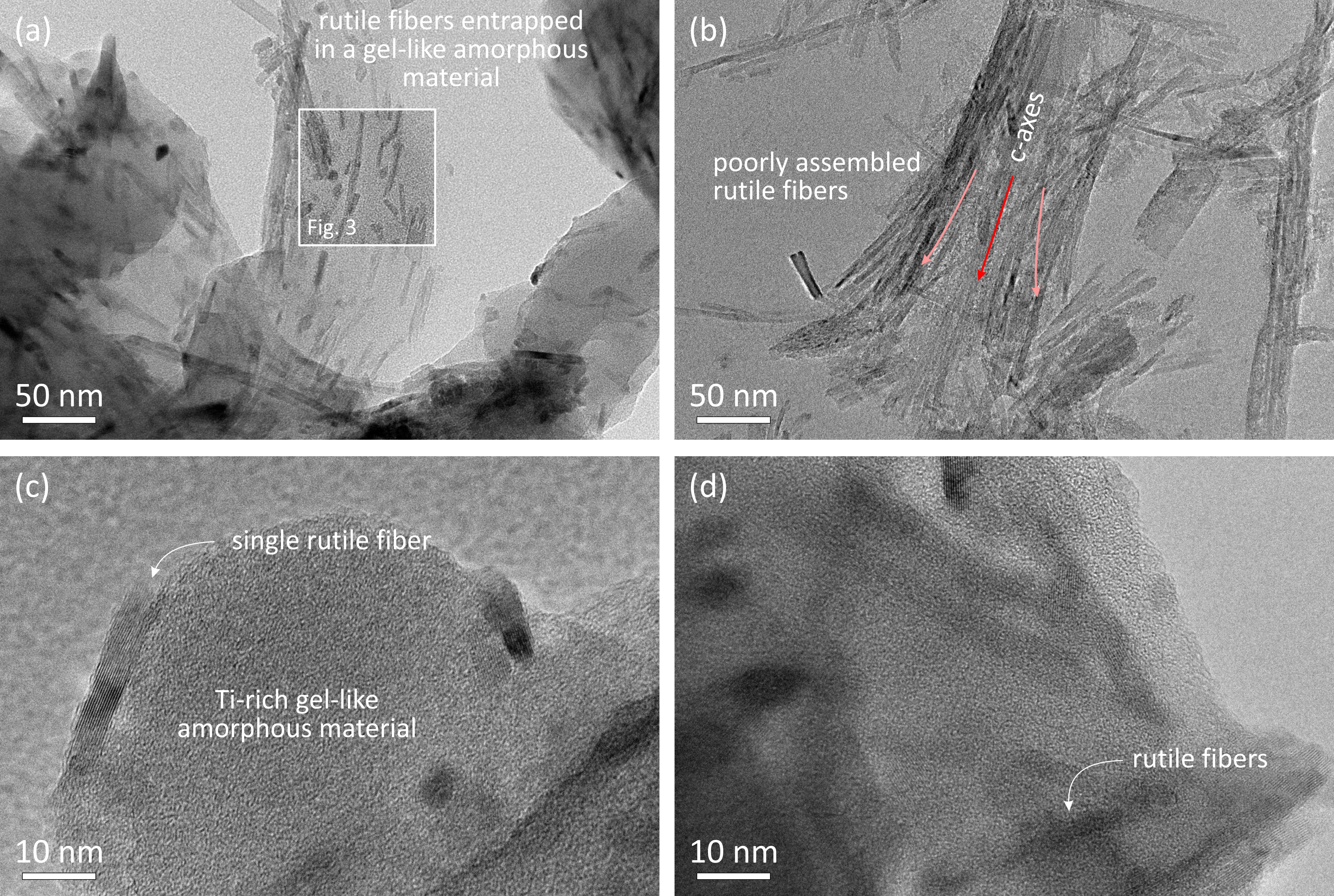


**Figure S3|Nucleation of fibrous rutile from Ti-rich amorphous precursor**. (**a**) Gel-like material present after short time of HT treatment contains isolated equilibrium shape rutile fibers that formed by condensation reactions (*see* Fig. 7). Precipitated fibers are generally disordered, however in some parts they appear to be aligned in parallel, regardless the considerable distance (~ 20 nm) between them (for details on short-range self-assembly *see* Fig. 4). (**b**) Onset of fiber assembly. Several rutile fibers in a cluster show a general alignment along the crystallographic c-axis. (**c**, **d**) Details of amorphous Ti-precursor with isolated rutile fibers, produced through oxolation reaction. Darker sections correspond to vertically aligned rutile fibers embedded in a gel-like material.

**Table S2 | Calculations of partial charge increase in various Ti-complexes formed by protonation and olation reaction:** Calculated partial charges of Ti-complexes formed by the olation reaction between different initially protonated complexes (depending on the medium acidity), using a model proposed by Livage (1988)^3^ *et al*. With olation reaction and formation of positively charged complexes due to protonation, partial charges of OH^-^ ligands are increasing, therefore losing their nucleophilic power. If initially complexes are not protonated (0 complexes) or slightly protonated (1+ complexes) olation can start easily and continue until OH^-^ ligands on titanium cation remain negatively charged. However, if initially complexes are highly charged, olation reaction is stopped when a dimer is formed in the case of 2+ complexes or not possible for 3+ complexes.

| Complexes by olation | ***δ(Ti)*** | ***δ(O)*** | ***δ(H)*** | ***δ(OH)*** | ***δ(H_2_O)*** |
| --- | --- | --- | --- | --- | --- |
| **[Ti(OH)_4_(OH2)_2_]^0^** | 0.753 | -0.394 | 0.2014 | -0.193 | 0.0088 |
| [Ti_2_(OH)_8_(OH2)_2_]^0^ | 0.755 | -0.393 | 0.202 | -0.191 | 0.011 |
| [Ti_10_(OH)_40_(OH2)_20_]^0^ | 0.753 | -0.394 | 0.201 | -0.193 | 0.008 |
| **[Ti(OH)_3_(OH2)_3_]^1+^** | 0.821 | -0.353 | 0.255 | -0.098 | 0.157 |
| [Ti_2_(OH)_6_(OH2)_4_]^2+^ | 0.837 | -0.343 | 0.268 | -0.075 | 0.193 |
| [Ti_10_(OH)_30_(OH2)_12_]^10+^ | 0.857 | -0.331 | 0.284 | -0.047 | 0.237 |
| [Ti_20_(OH)_60_(OH2)_22_]^20+^ | 0.86 | -0.329 | 0.286 | -0.043 | 0.243 |
| [Ti_40_(OH)_120_(OH2)_42_]^40+^ | 0.861 | -0.328 | 0.287 | -0.041 | 0.246 |
| [Ti_450_(OH)_1350_(OH2)_452_]^450+^ | 0.863 | -0.327 | 0.289 | -0.038 | 0.251 |
| **[Ti(OH)_2_(OH2)_4_]^2+^** | 0.88 | -0.316 | 0.302 | -0.014 | 0.288 |
| [Ti2(OH)_4_(OH2)_6_]^4+^ | 0.908 | -0.299 | 0.324 | **0.025** | **0.349** |
| **[Ti(OH)(OH2)_5_]^3+^** | 0.932 | -0.285 | 0.343 | **0.058** | **0.401** |
| [Ti2(OH)_2_(OH2)_8_]^6+^ | 0.968 | -0.263 | 0.371 | **0.108** | **0.479** |

**Supporting References:**

1. Pauling, L., Pauling, P. *Chemistry* (W.H. Freeman and Company: San Francisco, 1975).
2. Cao, G. *Nanostructures & Nanomaterials Synthesis, Properties & Applications* (Imperial College Press: London UK, 2004).
3. Livage, J., Henry, M., Sanchez, C. Sol-gel chemistry of transition metal oxides. *Prog. Solid St. Chem.* **18,** 259-341 (1988).
